# Atrial Fibrillation and Transvenous Lead Extraction—A Comprehensive Subgroup Analysis of the GermAn Laser Lead Extraction RegistrY (GALLERY)

**DOI:** 10.3390/medicina58111685

**Published:** 2022-11-21

**Authors:** Da-Un Chung, Simon Pecha, Heiko Burger, Omar Anwar, Christian Eickholt, Herbert Nägele, Hermann Reichenspurner, Nele Gessler, Stephan Willems, Christian Butter, Samer Hakmi

**Affiliations:** 1 Department of Cardiology and Critical Care Medicine, Asklepios Klinik St. Georg, Lohmuehlenstrasse 5, 20099 Hamburg, Germany; 2 Department of Cardiovascular Surgery, University Heart & Vascular Center Hamburg, 20251 Hamburg, Germany; 3 DZHK (German Centre for Cardiovascular Research), Partner Site Hamburg/Kiel/Lubeck, 20249 Hamburg, Germany; 4 Department of Cardiac Surgery, Kerckhoff Klinik, 61231 Bad Nauheim, Germany; 5 Department of Internal Medicine & Cardiology, Hospital Itzehoe, 25524 Itzehoe, Germany; 6 Department for Cardiac Insufficiency and Device Therapy, Albertinen-Hospital, 22457 Hamburg, Germany; 7 Department of Cardiology, Heart Center Brandenburg Bernau, 16816 Neuruppin, Germany

**Keywords:** transvenous lead extraction, atrial fibrillation, device complications, lead management, endocarditis

## Abstract

*Background*: Atrial fibrillation is the most common arrhythmia and has been described as driver of cardiovascular morbidity and risk factor for cardiac device-related complications, as well as in transvenous lead extraction (TLE). *Objectives*: Aim of this study was to characterize the procedural outcome and risk-factors of patients with atrial fibrillation (AF) undergoing TLE. *Methods*: We performed a subgroup analysis of all AF patients in the GALLERY (GermAn Laser Lead Extraction RegistrY) database. Predictors for all-cause mortality were assessed. *Results*: A total number of 510 patients with AF were identified with a mean age of 74.0 ± 10.3 years. Systemic infection (38.4%) was the leading cause for TLE, followed by local infection (37.5%) and lead dysfunction (20.4%). Most of the patients (45.9%) presented with pacemaker systems to be extracted. The total number of leads was 1181 with a 2.3 ± 0.96 leads/patient. Clinical procedural success was achieved in 97.1%. Occurrence of major complications was 1.8% with a procedure-related mortality of 1.0%. All-cause mortality was high with 5.9% and septic shock being the most common cause. Systemic device infection (OR: 49.73; 95% CI: 6.56–377.09, *p* < 0.001), chronic kidney disease (CKD; OR: 2.67; 95% CI: 1.01–7.03, *p* = 0.048) and a body mass index < 21 kg/m^2^ (OR: 6.6; 95% CI: 1.68–25.87, *p* = 0.007) were identified as independent predictors for all-cause mortality. *Conclusions*: TLE in AF patients is effective and safe, but in patients with systemic infection the mortality due to septic shock is high. Systemic infection, CKD and body mass index <21 kg/m^2^ are risk factors for death in patient with AF undergoing TLE.

## 1. Introduction

In 2019 atrial fibrillation affected over 60 million adults around the globe and remains one of the strongest contributors for cardiovascular morbidity and mortality in the world [1]. Extrapolations on current trends suggest, that the prevalence of atrial fibrillation is going to increase globally by >60% until 2050 [2]. In light of the continuously increasing numbers of cardiac implantable electronic devices (CIED) [3], the overlap between patients with atrial fibrillation and indwelling CIED is likely to further increase as well. The lifetime risk of developing device complications, especially CIED-related infections (CDRI), remains high with a cumulative probability of 11.7% in 25 years and creates a pronounced financial burden on our healthcare systems annually [4,5]. The risk and incidence of device-related infections is particularly increased for complex devices, such as implantable cardioverter defibrillators (ICD) or cardiac resynchronization therapy (CRT) devices, which have become a mainstay for heart failure therapy [6,7]. The prevalence of atrial fibrillation in heart failure patients has been described to range from 10.1–49.8% with increasing New York Heart Association (NYHA) class [8]. Transvenous lead extraction (TLE) has evolved into a crucial therapeutic pillar for patients with CIED-related complications and represents a Class I indication in international consensus statements in cases of CDRI [9,10], where timely extraction seems to be paramount to ensure patient survival [11]. Atrial fibrillation has been described as independent risk factor for systemic CDRI in a recent large study [12], but there is little known about procedural outcome and risk factors of patients with atrial fibrillation undergoing TLE. The aim of this study was to characterize AF patients enrolled into the GALLERY for TLE for any cause and analyze determinants of procedural success and failure, as well as predictors for all-cause mortality.

## 2. Methods

### 2.1. Patients Population & Study Design

The GALLERY enrolled all patients that underwent laser lead extraction from 24 participating centers between January 2013 and March 2017. The registry included 2524 patients with 6117 leads treated. This study was designed as post hoc subgroup analysis of all patients in the GALLERY including all patients with atrial fibrillation recorded on ECG at admission. The detailed description of data collection and maintenance, as well as the study design of the GALLERY has already been published in the main manuscript [13]. This study complies with the Declaration of Helsinki and the study protocol was approved by the ethics committee of the state medical board Hamburg (Ärztekammer Hamburg; reference number: WF-026/17).

### 2.2. Definitions

All definitions pertaining TLE procedure, tools and techniques utilized, as well as procedural complications and outcomes in this study, adhere to the specifications previously published and described by the Heart Rhythm Society (HRS) and European Heart Rhythm Association (EHRA) expert consensus statements on lead extraction [9,10]. Complete lead removal was defined as the extraction of all targeted lead material from the vascular space. Incomplete lead removal was defined as remaining or abandoned leads or lead fragments (>4 cm) in the patient’s body by the end of the procedure. Complete procedural success was determined by removal of all targeted lead material with the absence of any permanently disabling complication or procedure-related death. Clinical procedural success was defined as retention of small lead fragments that do not negatively impact the goals of the procedure, in the absence of any permanently disabling complication or procedure-related death. Procedural failure was defined as the inability to achieve complete procedural- or clinical success, and/or the development of any permanently disabling complication or procedure-related death. Major procedure-related complications were defined as complications which, were either life-threatening or resulted in death or any other significant persisting disabling condition. A minor complication was every procedure-related undesired event, leading to a medical or minor procedural intervention, without persistent or significant influence on patient’s functional capacity. Procedure-related death was defined as any death that occurred during the extraction procedure or was directly or indirectly associated with a procedural complication. In-hospital mortality was defined as any death (cardiac or non-cardiac) that occurred during the hospital stay, irrespective of its relation to the procedure.

### 2.3. Study Objectives

This study aimed at analyzing all patients with atrial fibrillation undergoing TLE regarding patient characteristics, device- and lead demographics, as well as procedural outcomes and adverse events. Furthermore, the study sought to identify specific independent risk factors, to predict all-cause mortality in patients with atrial fibrillation undergoing TLE.

### 2.4. Lead Extraction Technique

All procedures were performed by fluoroscopic guidance in an operating room under general anaesthesia. Continuous arterial blood pressure monitoring was performed. Transoesophageal echocardiography was used to guide the procedure. All patients were prepared for emergent sternotomy with a primed cardiopulmonary bypass circuit on standby. Leads were dissected from the surrounding tissue and the sleeves were removed. Lead locking devices (LLD) were placed. Laser lead extraction was conducted using GlideLight 80 Hz or SLS II 40 Hz laser sheaths Sheath sizes included 14 or 16 French sheaths. Use of additional powered- or non-powered extraction sheaths (Mechanical rotational sheaths, non-powered extraction sheaths, snares or other tools) was allowed, if at least one laser sheath was used during the extraction procedure.

### 2.5. Statistics

Continuous variables are expressed as mean ± standard deviation (SD) for normal distributions and median and interquartile range (IQR) for non-gaussian distributions. Categorial variables are shown as counts and percentages. Categorical variables between groups were compared using χ^2^-test or Fisher’s exact test, in case of small sample sizes (<5 counts per cell). Univariate and multivariate logistic regression analysis was used to determine independent predictors for in-hospital mortality and procedural failure. Predictor variables that reached statistical significance in univariate analysis and additional clinically relevant covariates were included into the multivariate analyses. A 2-tailed *p*-value of <0.05 was considered as statistically significant. Statistical analysis was performed using Prism 8 (GraphPad Software, San Diego, CA, USA) and IBM SPSS 25.0 statistical software package (IBM, Armonk, NY, USA).

## 3. Results

### 3.1. Patient & Device Characteristics

**Figure 1:**
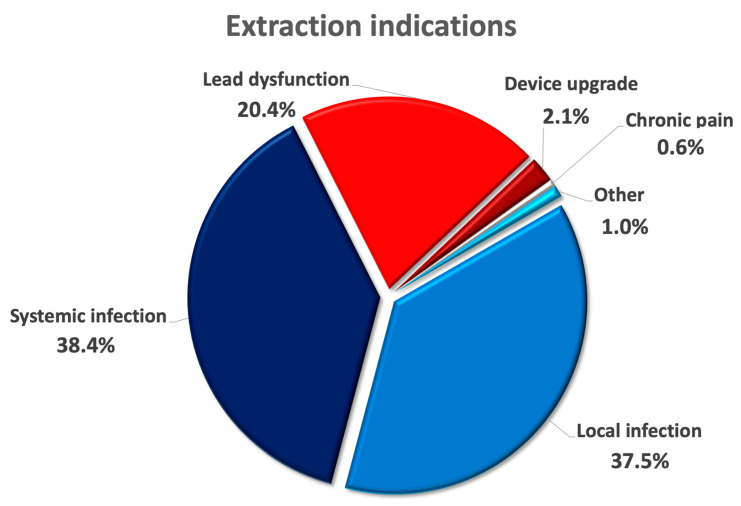
Pie chart depicting the various extraction indications of patients with atrial fibrillation undergoing transvenous lead extraction in percent.

A total number of 510 patients with atrial fibrillation (20.2% of the GALLERY) were identified with a mean age of 74.0 ± 10.3 years and 22.2% female patients. Mean body mass index (BMI) was 27.4 ± 4.8 kg/m^2^ and the proportion of patients with severely reduced left-ventricular ejection fraction (LVEF) was 26.3%. Arterial hypertension was present in 77.3% of cases, along with other comorbidities such as coronary artery disease (52.4%), diabetes mellitus (38.4%), chronic kidney disease (40.2%) and history of previous cardiac surgery (27.8%). The majority of patients presented with an infectious extraction indication (Local infections: 37.5%; systemic infections: 38.4%), followed by 20.4% of patients with lead dysfunction and 2.1% with due to device upgrade (Figure 1). The distribution of extracted devices was pacemakers in 45.9%, ICD in 31.9% and CRT devices in 22.2% of patients. The total number of leads was 1181 with a mean lead-to-patient ratio of 2.3 ± 0.96 and a median age of the oldest lead of 106.5 [66; 154] months. There were 34.1% of patients with right-sided leads and 32.0% of patients with abandoned leads (see also Table 1).

### 3.2. Procedure and Outcome

**Figure 2:**
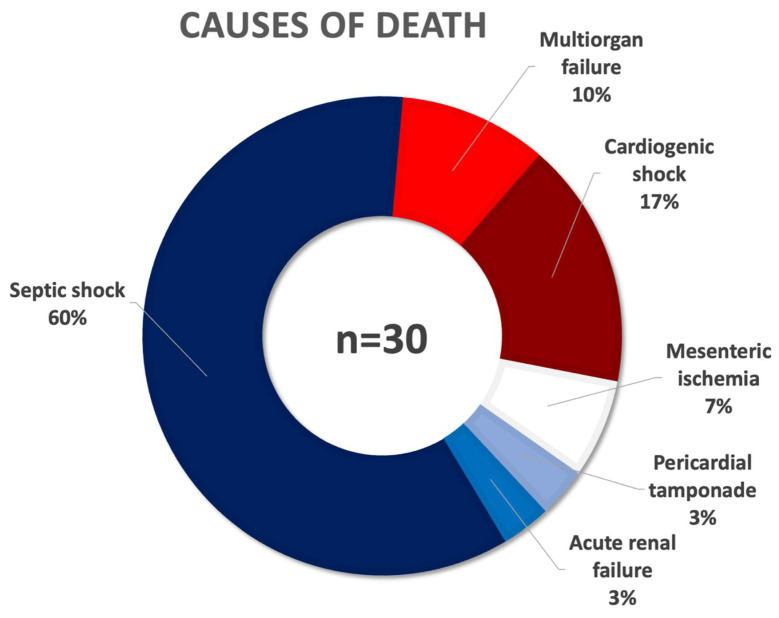
Ring chart showing the different causes of death in percent of all patient in-hospital deaths (*n* = 30).

The median hospital stay was 10 [6; 18] days with a median postoperative stay of 7 [4; 14] days. The median procedural time was 77.5 [51.75; 120] minutes. Overall complication rate was 3.6% with rates of 1.8% and 1.8% minor and major complications, respectively. Most common minor complication was pocket hematoma in 7 cases (1.4%), whereas laceration of the superior vena cava (SVC) or cavo-atrial junction in 3 cases (0.6%) and perforation of the right atrium or ventricle in 3 cases (0.6%) were the leading major complications. Two more patients (0.4%) suffered pericardial tamponade, while one patient (0.2%) had hemothorax that required drainage. Five patients succumbed to the severity of their complications, resulting in a procedure-related mortality of 1.0%. Complete procedural success and clinical procedural success were achieved in 92.4% and 97.1% of extractions, respectively. All-cause in-hospital mortality was 5.9% (*n* = 30), which was mainly driven by septic shock in 18 cases (3.6%). More details on procedural data and outcome are listed in Table 2 and shown in Figure 2.

### 3.3. Risk Factors for All-Cause Mortality

In order to identify predictors for all-cause mortality in patients with atrial fibrillation undergoing TLE, we conducted univariate and multivariate logistic regression with pre-selected candidate variables. Univariate regression (data not shown) revealed a BMI < 21 kg/m^2^ (OR: 4.4; 95% CI: 1.64–11.61; *p* = 0.003), chronic kidney disease (OR: 3.75; 95% CI: 1.68–8.37; *p* = 0.001) and systemic infection (OR: 54.35; 95% CI: 7.34–402.54; *p* < 0.001) as risk factors for all-cause in-hospital mortality. The presence of coronary artery disease (CAD) showed a trend to being a risk factor, but failed to reach statistical significance (OR: 2.22; 95% CI: 0.99–4.95; *p* = 0.051) in univariate analysis. In the following multivariate analysis and adjustment for each other, a BMI < 21 kg/m^2^ (OR: 6.6; 95% CI: 1.68–25.87; *p* = 0.007), chronic kidney disease (OR: 2.67; 95% CI: 1.01–7.03; *p* = 0.048) and systemic infection (OR: 49.73; 95% CI: 6.56–377.09; *p* < 0.001) remained independent predictors for all-cause mortality for patient with atrial fibrillation undergoing transvenous lead extraction (Table 3).

## 4. Discussion

In this GALLERY subgroup analysis of patients with atrial fibrillation undergoing transvenous lead extraction, we observed a high efficacy of transvenous lead extraction therapy. Clinical procedural success rate was achieved in 97.1% of patients. We additionally observed a low rate of procedure-related major complications (1.8%) as well as a low procedure-related mortality (1.0%). Procedure-related major complications are in line with results from previously published large lead extraction registries like ELECTRa with a major complication rate of 1.7% [14] or other studies using mechanical rotational sheaths, which have shown complication rates ranging between 0% and 1.5% [15,16,17,18]. Previous laser lead extraction studies have reported major complication rates between 0.9 and 2.5% [19,20,21,22,23]. Procedure-related mortality has also been comparable to previously published lead extraction studies like the “US total laser lead extraction experience” by Byrd et al. (0.8% procedure-related mortality) the Plexes trial (0.41% procedure related mortality) [24,25], or the PROMET Evolution subgroup (0.4% procedure-related mortality) [15]. Nevertheless, the overall in-hospital mortality rate was rather high with 5.9%, mainly due to non-procedure-related septic shock in patients with systemic CDRI. The in-hospital mortality in this subgroup analysis is even higher as in the overall GALLERY cohort, where an in-hospital mortality rate of 3.56% was observed [13]. This high rate of in-hospital mortality was mainly driven by the occurrence of septic shock in patients with systemic infection, which was the leading cause of death in this subgroup analysis. The presence of systemic lead related infection has been shown to be an independent predictor for in-hospital mortality in previous lead extraction studies [7,13]. The high number of patients with systemic infection in this AF subgroup (38.4%) might have contributed to the higher rate of non-procedure-related in-hospital mortality. In other large lead extraction studies like Plexes or ELECTRa the percentage of patients with systemic infection was considerably lower with 15% and 19.2%, respectively [14,25]. Complex devices, such as ICD or CRT, which made up 54.1% of all extracted devices in this subgroup (Table 1), have been previously described as risk factor for CDRI [6]. The presence of AF has been shown to be a risk factor systemic CDRI in a previous GALLERY subgroup analysis of patients with systemic device related infection, which might be explained by the association between clinically significant pocket hematomas under therapeutic anticoagulation and the increased long-term risk of CDRI [12,26]. However, predictors and mechanisms for higher mortality risk in AF patients undergoing transvenous lead extraction remain unclear. In this analysis of patients with AF, we investigated predictors for mortality in this subgroup. Besides systemic infection, which was the strongest predictor for death, chronic kidney disease and lower BMI were independent predictors for non-procedure related mortality. Since the prevalence of atrial fibrillation increases with age, the median age of patients in this subgroup analysis (74.0 ± 10.3) was remarkably higher than in the overall GALLERY cohort [13] and in other large registries like PROMET or ELECTRa [14,15]. Advanced age has been a risk factor for mortality in previous lead extraction registries like PROMET [15]. In patients with atrial fibrillation the incidence of comorbidities like arterial hypertension, heart failure, coronary artery disease and valvular heart disease are significantly increased [1,27]. Accordingly, comorbidities like coronary artery disease (52.4% vs. 42.9%), chronic kidney disease (40.2% vs. 31.1%), diabetes mellitus (38.4% vs. 31.2%) and arterial hypertension (77.3% vs. 70.5%) were more pronounced in the AF subgroup than in the overall GALLERY population [13], representing a considerably sicker patient cohort. Interestingly, the number of heart failure patients with highly reduced LVEF was comparable between the overall GALLERY cohort and this AF subgroup analysis. There is a well-known impact of atrial fibrillation on mortality in the general population with an annual mortality rate between 1.4% and 7.0% in patients with AF according to several studies [1,2,28,29]. If this mortality is associated with the atrial fibrillation itself, or its related comorbidities remains unclear. To which extent the presence of atrial fibrillation has contributed to a higher in-hospital mortality in our subgroup cannot be clarified in our study. Most likely, the co-morbidities together with the high percentage of systemic infection might have triggered the high rate of in-hospital mortality. Clinical procedural success rates were comparable to previously published large studies and registries like Plexes, PROMET [15] and ELECTRa with success rates of 97.7%, 97.0% and 96.7%, respectively [14,25]. Although in our subgroup analysis the median lead implant duration was remarkably longer with 106.5 months in comparison to abovementioned studies (Plexes: 65.0 months, PROMET: 74.0 months, ELECTRa: 76.8 months). This shows the efficacy of the transvenous lead extraction procedure itself.

However, even in patients with successful lead extraction, the mortality remains high in presence of systemic infection [12]. Therefore, different strategies need to be applied to treat these critically ill patients. Since time till diagnosis and treatment plays an important role in patients with systemic infection [11], the awareness for cardiac device related infections needs to be sharpened for primary care physicians and referral centers to get the patients at an early stage into a specialized lead extraction center.

## 5. Conclusions

Transvenous lead extraction in patients with atrial fibrillation is effective and safe, but the mortality due to septic shock is high. Systemic infection, chronic kidney disease and body mass index <21 kg/m^2^ are risk factors for death in patient with atrial fibrillation undergoing lead extraction.

## 6. Limitations

The main limitation of our study is the retrospective nature of the analysis with the possibility of selection- and detection bias. Despite greatest efforts to keep biases to a minimum by employing standardized definitions and a predefined case report form, unknown influential factors due to differences in therapeutic decisions of individual physicians involved in this study cannot be ruled out. The inclusion criterion for atrial fibrillation was solely based in the rhythm documented in the admission ECG, thus patients with paroxysmal atrial fibrillation and sinus rhythm at admission were possibly not recorded. Since only high-volume referral centers with >20 TLE cases annually, along with a more complex cross-section of extraction cases were involved, data on patient characteristics and procedural outcomes might be skewed and not generalizable, but applicable for other large centers with TLE expertise. The fact that the GALLERY mainly utilized laser sheaths for extraction might also have influenced our results and further studies including a wider variety of extraction techniques should be encouraged.

## Figures and Tables

**Table 1:** Patient and lead characteristics.

	Atrial Fibrillation (*n* = 510)
Mean age, years ± SD	74.0 ± 10.3
Female sex, *n* (%)	113 (22.2)
Mean BMI, kg/m^2^ ± SD	27.4 ± 4.8
LVEF ≤ 30%, *n* (%)	134 (26.3)
Arterial hypertension, *n* (%)	394 (77.3)
Coronary artery disease, *n* (%)	267 (52.4)
Diabetes mellitus, *n* (%)	196 (38.4)
Chronic kidney disease, *n* (%)	205 (40.2)
Previous heart surgery, *n* (%)	142 (27.8)
Extraction indication	
Device infection, *n* (%)	387 (75.9)
- Local infection, *n* (%)	197 (37.5)
- Systemic infection, *n* (%)	196 (38.4)
Lead dysfunction, *n* (%)	104 (20.4)
System upgrade, *n* (%)	11 (2.1)
Chronic pain, *n* (%)	3 (0.6)
Other, *n* (%)	5 (1.0)
Extracted devices	
Pacemaker, *n* (%)	234 (45.9)
ICD, *n* (%)	163 (31.9)
CRT, *n* (%)	113 (22.2)
Leads	
Total number of leads, *n*	1181
Leads per patient, *n* ± SD	2.3 ± 0.96
Median age of the oldest leads, months [IQR]	106.5 [66; 154]
Patients with abandoned leads, *n* (%)	163 (32.0)
Patients with right-sided leads, *n* (%)	174 (34.1)

Values are expressed as mean ± SD or counts (*n*) and percentages (%), BMI: body mass index, ICD: implantable cardioverter-defibrillator; IQR: interquartile range; SD: standard deviation.

**Table 2:** Procedural data.

	Atrial Fibrillation (*n* = 510)
Median hospital stay, days [IQR]	10.0 [6; 18]
Median postoperative stay, days [IQR]	7.0 [4; 14]
Median procedural time, minutes [IQR]	77.5 [51.75; 120]
Overall complications, *n* (%)	18 (3.6)
- Major complications, *n* (%)	9 (1.8)
- Minor complications, *n* (%)	9 (1.8)
Complete procedural success, *n* (%)	471 (92.4)
Clinical procedural success, *n* (%)	496 (97.2)
Procedure-related mortality, *n* (%)	5 (1.0)
All-cause mortality, *n* (%)	30 (5.9)
- Septic shock, *n* (%)	18 (3.6)
- Multiorgan failure (not further specified), *n* (%)	3 (0.6)
- Cardiogenic shock, *n* (%)	5 (1.0)
- Pericardial tampoande, *n* (%)	1 (0.2)
- Mesenteric ischemia, *n* (%)	2 (0.4)
- Acute renal failure, *n* (%)	1 (0.2)

Values are expressed as median with interquartile ranges (IQR) or counts (*n*) and percentages (%), ICD: implantable cardioverter-defibrillator, VF: ventricular fibrillation, VT: ventricular tachycardia.

**Table 3:** Multivariate regression analysis—predictors for all-cause mortality.

Variables	OR	95% CI	*p*-Value
BMI < 21 kg/m^2^	7.14	1.91–26.70	0.004
BMI ≥ 35 kg/m^2^	0.41	0.05–3.47	0.410
Arterial hypertension	0.84	0.29–2.41	0.740
Chronic kidney disease	2.78	1.08–7.16	0.034
Coronary artery disease	1.62	0.63–4.14	0.316
Diabetes mellitus	0.94	0.38–2.32	0.893
Right-sided leads	1.06	0.41–2.77	0.900
LVEF < 30%	0.95	0.36–2.53	0.923
Previous cardiac surgery	1.11	0.42–2.95	0.830
Abandoned leads	1.32	0.44–3.92	0.621
≥4 leads in situ	1.06	0.27–4.17	0.936
Lead Age ≥ 10 years	0.72	0.27–1.92	0.507
Systemic infection	50.66	6.70–382.9	<0.001

*p*-values < 0.05 were considered statistically significant; BMI: body mass index; CI: confidence interval; LVEF: left ventricular ejection fraction; OR: odds ratio.

## Data Availability

All data pertaining this study will be made available upon reasonable request by the corresponding author.

## Appendix A

GALLERY (GermAn Laser Lead Extraction RegistrY) investigators: Simon Pecha, Heiko Burger, Da-Un Chung, Viviane Möller, Tomas Madej, Alaa Maali, Brigitte Osswald, Raffaele De Simone, Nadeja Monsefi, Virgilijus Ziaukas, Stefan Erler, Hamdi Elfarra, Mathias Perthel, Mahmoud S. Wehbe, Naser Ghaffari, Tim Sandhaus, Henning Busk, Jan D. Schmitto, Volker Bärsch, Jerry Easo, Marc Albert, Hendrik Treede, Herbert Nägele, Dieter Zenker, Yasser Hegazy, Donja Ahmadi, Nele Gessler, Wolfgang Ehrlich, Gabriele Romano, Michael Knaut, Hermann Reichenspurner, Stephan Willems, Christian Butter, and Samer Hakmi.
